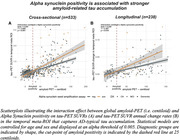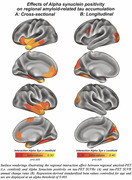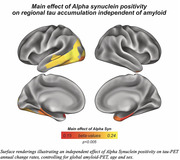# Alpha Synuclein co‐pathology is associated with faster Ab‐related tau accumulation in Alzheimer's disease

**DOI:** 10.1002/alz.093961

**Published:** 2025-01-09

**Authors:** Sebastian Niclas Roemer, Amir Dehsarvi, Anna Steward, Anna Dewenter, Julia Pescoller, Fabian Wagner, Johannes Levin, Matthias Brendel, Günter Höglinger, Nicolai Franzmeier

**Affiliations:** ^1^ Department of Neurology, University Hospital, LMU, Munich, Bavaria Germany; ^2^ Institute for Stroke and Dementia Research (ISD), University Hospital, LMU, Munich, Bavaria Germany; ^3^ Institute for Stroke and Dementia Research (ISD), LMU University Hospital, Munich, Munich (Bavaria) Germany; ^4^ Department of Neurology, LMU University Hospital, LMU Munich, Munich Germany; ^5^ Department of Neurology, Klinikum der Ludwig‐Maximilians Universität München, Munich Germany; ^6^ Munich Cluster for Systems Neurology (SyNergy), Munich, Bavaria Germany

## Abstract

**Background:**

Lewy body pathology consisting of aggregated alpha‐Synuclein (a‐Syn) is the hallmark pathology in Parkinson’s disease, yet a‐Syn aggregates are also commonly observed post‐mortem as a co‐pathology in Alzheimer’s disease (AD) patients. Preclinical research has shown that a‐Syn can amplify Ab‐associated tau seeding and aggregation, hence a‐Syn co‐pathology may contribute to the Ab‐induced progression of tau pathology in AD. To address this, we combined a novel CSF‐based RT‐QuIC seed‐amplification assay to determine a‐Syn positivity, with PET‐neuroimaging in a large patient cohort ranging from cognitively normal to dementia, to determine whether a‐Syn co‐pathology accelerates Ab‐driven tau accumulation.

**Method:**

In 261 Ab‐positive vs. 272 Ab‐negative subjects ranging from cognitively normal to dementia we employed amyloid‐PET, Flortaucipir tau‐PET and a CSF‐based a‐Syn RT‐QuIC assay for in vivo detection of abnormal a‐Syn aggregation. A subset of 136 Ab‐positive vs. 102 Ab‐negative subjects had longitudinal tau‐PET across ∼2.5years. Using linear regression, we tested whether a‐Syn positivity was linked to stronger Ab‐related tau aggregation (i.e. interaction a‐Syn x amyloid‐PET on tau‐PET).

**Result:**

Prevalence of a‐Syn positivity rose across increasing clinical severity and was particularly pronounced in Ab+ (i.e. CN/MCI/Dementia=20/23/47%) vs. Ab‐ subjects (i.e. CN/MCI/Dementia=15/10/29%), suggesting that a‐Syn co‐pathology is more common in clinically advanced AD (chi‐squared‐test, p<0.001). When testing the interaction between a‐Syn and global amyloid‐PET, we found that a‐Syn positivity was associated with stronger Ab‐related tau deposition (Figure 1A, p<0.001), and faster Ab‐related tau accumulation rates (Figure 1B, p=0.010) in typical tau vulnerable brain regions (i.e. temporal meta ROI), adjusting for age and sex. Regional analyses confirmed that higher regional amyloid‐PET was associated with stronger temporal‐lobe tau deposition (Figure 2A) and faster tau accumulation in downstream regions (Figure 2B) in a‐Syn positive individuals. In addition, there was an independent effect of a‐Syn positivity on faster temporal lobe tau accumulation rates controlling for age, sex and global amyloid, suggesting that a‐Syn may also independently contribute to tau aggregation (Figure 3).

**Conclusion:**

a‐Syn co‐pathology as detected by CSF seed‐amplification assays is more common at clinically advanced AD and related to faster Ab‐related tau aggregation. This suggests that a‐Syn co‐pathology may actively contribute to AD‐related tau accumulation and therefore contribute to dementia development.